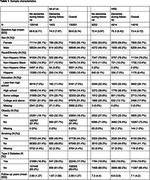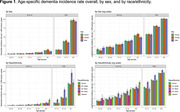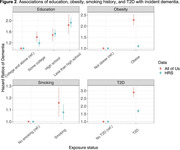# Comparison of dementia incidence and risk factor associations in All of Us against the nationally representative Health and Retirement Study

**DOI:** 10.1002/alz.091815

**Published:** 2025-01-09

**Authors:** Jingxuan Wang, Ruijia Chen, Eleanor Hayes‐Larson, Elizabeth Rose Mayeda, Minhyuk Choi, Sarah F Ackley, M. Maria Glymour

**Affiliations:** ^1^ University of California, San Francisco, San Francisco, CA USA; ^2^ Boston University, Boston, MA USA; ^3^ UCLA Fielding School of Public Health, University of California, Los Angeles, CA USA; ^4^ University of California San Francisco, San Francisco, CA USA; ^5^ Boston University School of Public Health, Boston, MA USA

## Abstract

**Background:**

The All of Us (AoU) Research Program, an ongoing large‐scale research initiative enrolling diverse groups across the U.S offers a valuable opportunity for dementia research. It is vital to assess if AoU findings are applicable to the general population. We compared dementia incidence and risk factor associations in AoU to those in a population‐based cohort, the Health and Retirement Study (HRS).

**Method:**

AoU participants aged ≥ 60 without a dementia diagnosis at baseline (N = 130,551, mean age = 69.6) were included. We identified all‐cause dementia cases by ICD codes from linked EHRs. We included HRS participants aged ≥ 70 from 2000 to 2016 who had no dementia status before baseline (N = 14,016, mean age = 73.4). Dementia status was determined at each wave using a validated algorithm (Expert Model). We calculated age‐specific dementia incidence rates overall and by sex and race/ethnicity. We used Cox models to evaluate associations of education, obesity (body mass index ≥ 30), smoking history (ever vs not), and type 2 diabetes (T2D) with incident dementia, adjusting for age, sex, and race/ethnicity.

**Result:**

1,403 dementia cases accrued during a mean of 2.6 years follow‐up in AoU compared to 4,814 dementia cases during a median of 7.1 years follow‐up in HRS (Table 1). Age‐specific dementia incidence rates over 70 years were lower in AoU compared with HRS, with more pronounced differences at older ages (Figure 1). Risk factor associations showed larger relative effect sizes in AoU, except for education, which showed similar associations (Figure 2). For example, the hazard ratio for T2D and dementia in AoU (HR = 2.89, 95%CI = 2.59‐3.23) was larger than in HRS (HR = 1.69, 95%CI = 1.57‐1.82, Figure 2).

**Conclusion:**

Dementia incidence rates were much lower in AoU than HRS, consistent with prior work showing a healthy volunteer bias for study enrollment. This may also represent underdiagnosis of dementia in the EHR or overdiagnosis in HRS. Associations between risk factors and dementia were similar or stronger in AoU compared to the HRS. Future studies should approach the generalization of AoU study findings to the US adult population with caution.